# Sanatoria for Consumption

**Published:** 1901-08-17

**Authors:** 


### The Hospital

A Journal of

The Medical Sciences and Hospital Administration.

Vol. XXX.—No. 777] Edited by Sir Henry Burdett, K.C.B., and Solomon C. Smith, M.D., M.R.C.P. [August 17, 1901.

### SANATORIA FOR CONSUMPTION.

It can hardly be doubted that one effect of the recent Congress on Tuberculosis will be to give a great impulse to the establishment of nursing homes, or of institutions styled "sanatoria," for the reception of patients suffering from pulmonary phthisis; and it is somewhat to be feared that expectation has been unduly excited, as far as the public are concerned, with regard to the nature and the permanence of the results which these establishments are calculated to secure. People of undisciplined imagination, excited by a smattering of knowledge, or, more properly, of information, are proclaiming in all directions that consumption is a curable malady, and that its cure is to be brought about by the comparatively simple method of going to live in a "sanatorium," situate, if possible, in some foreign locality the name of which those who resort to it are unable to pronounce, and controlled by some doctor of whose claims to confidence they are wholly unable to form any valid or trustworthy opinion. As long as there are open doors and windows, or merely apertures where doors and windows should be, and as long as "forced feeding" and exercise are controlled, not by the requirements of the patient, but by the "rule" of the particular establishment that may be selected, parents and friends remain in a fool's paradise of contentment, and declare, when the inevitable end is reached at last, that everything which was possible had been done.

The latest development of the belief is tending towards the establishment of "sanatoria" in this country, as charities for the benefit of the poor, and to these we wish all imaginable success. The patients admitted will certainly be placed under conditions more favourable than the generality of them would be able to command at home, and the results are likely to be observed without prejudice and to be recorded without partiality. Within a very few years, at least, every such sanatorium will be an object lesson to the residents in its neighbourhood with regard to the amount of real and permanent benefit which may reasonably be hoped for from its operations, or from the endeavour to arrest the progress of phthisis, to expel the bacilli from the system, and to bring about repair of the mischief which they have wrought, by the influence of pure air and of regimen alone. In precise proportion as the results fall short of perhaps too sanguine expectations, opportunities will be afforded of testing or demonstrating the usefulness, or the futility, as the case may be, of endeavours which would certainly be made to bring about a cure by other modes of treatment. The Sanatoria would thus provide conditions necessary for carrying out a series of therapeutical experiments, the results of which, whatever they might be, could not fail to be of real and permanent advantage to the public.

But, in the meanwhile, and during the existence of a period of excited public hopefulness as to the curability of consumption, we are confronted with a rapid springing up of "sanatoria" for paying patients as institutions owned by medical practitioners and conducted by them for the sake of gain, and this may well cause some anxiety as to the ethical problems involved. The first Lord Lytton many years ago aptly described one of the early "hydropathic" establishments as constituting "an ill-assorted union betwixt medicine and hotel-keeping." Now the hotel-keeper is exposed to temptations from which the physician would gladly be exempt, and may fairly claim to be judged by a different standard and from a different point of view. The physician not only should not be venal, but, like Cæsar's wife, he should not be exposed even to the suspicion of venality. At present, it may be truly said that no such suspicion exists. The institutions are few in number, they are owned by gentlemen of considerable professional distinction, and the eagerness to enter them is such that would-be patients are compelled to wait their turn. Is it reasonable to expect that these conditions will be permanent? Is it not more likely that the success of the institutions now existing will lead to the establishment of more, that these may be set up by men whose minds are directed rather towards realised profit than towards sound pathology, and that the subsidence of the present rush may lead to a general reduction of terms under the influence of competition, and to a state in which a few vacant beds may convert the expected profit into loss. Under such circumstances one may admit that the medical proprietors of such establishments might be exposed to many temptations. But so also would be the medical director of a sanatorium owned by non-medical proprietors. With the dread of dismissal, unless he could earn a dividend, constantly before him, he would be in an even reese plight. The real protection of the public against that insidious tendency to quackery which so often lurks under highly specialised modes of treatment lies in the honour of those members of our profession by whom such methods are applied; and to this we must trust it. It is by maintaining a high standard of science and of ethics that we shall best save the sanatorium treatment of consumption, and all other special methods which require a considerable capital expenditure, from that abyss of quackery into which they might so readily fall if run on purely commercial principles.

